# Distributed Reliable and Efficient Transmission Task Assignment for WSNs

**DOI:** 10.3390/s19225028

**Published:** 2019-11-18

**Authors:** Xiaojuan Zhu, Kuan-Ching Li, Jinwei Zhang, Shunxiang Zhang

**Affiliations:** 1 School of Computer Science and Engineering, Anhui University of Science and Technology, Huainan 554, China; xjzhu@aust.edu.cn (X.Z.); jwzhang@aust.edu.cn (J.Z.); sxzhang@aust.edu.cn (S.Z.); 2 Dept. of Computer Science and Information Engineering, Providence University, Taichung 43301, Taiwan

**Keywords:** wireless sensor networks, task assignment, distributed, reliable, energy-efficient

## Abstract

Task assignment is a crucial problem in wireless sensor networks (WSNs) that may affect the completion quality of sensing tasks. From the perspective of global optimization, a transmission-oriented reliable and energy-efficient task allocation (TRETA) is proposed, which is based on a comprehensive multi-level view of the network and an evaluation model for transmission in WSNs. To deliver better fault tolerance, TRETA dynamically adjusts in event-driven mode. Aiming to solve the reliable and efficient distributed task allocation problem in WSNs, two distributed task assignments for WSNs based on TRETA are proposed. In the former, the sink assigns reliability to all cluster heads according to the reliability requirements, so the cluster head performs local task allocation according to the assigned phase target reliability constraints. Simulation results show the reduction of the communication cost and latency of task allocation compared to centralized task assignments. Like the latter, the global view is obtained by fetching local views from multiple sink nodes, as well as multiple sinks having a consistent comprehensive view for global optimization. The way to respond to local task allocation requirements without the need to communicate with remote nodes overcomes the disadvantages of centralized task allocation in large-scale sensor networks with significant communication overheads and considerable delay, and has better scalability.

## 1. Introduction

Task assignment is an essential issue in wireless sensor networks (WSNs). In such multi-sensor systems, task allocation is based on the deadline and the priority of tasks, and different tasks are reasonably assigned to the sensor nodes for best perception performance. However, task allocation schemes that only aim at reducing energy consumption are not sufficient for several applications with high-reliability requirements. That is, whether tasks with high-reliability requirements in WSNs such as measurement, monitoring, and configuration can be completed in a timely manner depends on how the transmission tasks in WSNs can be reliably and efficiently assigned. As is known, energy-savings and high reliability are two conflicting goals, and it is thus challenging to assign high reliability and low energy tasks to appropriate nodes in WSNs.

In recent years, task priority assignment in WSNs has attracted the attention of researchers and satisfactory research results have been achieved. Most existing methods for practical applications are centralized algorithms, as the centralized task assignment method based on a single sink node still has the problem of poor reliability and scalability, so offline execution is selected due to computational complexity. Besides, the communication overhead and delay of task assignment will be significant in large-scale network scenarios or frequent network and dynamic changes. Therefore, it is very urgent to investigate novel distributed, reliable, and efficient task assignment methods for WSNs.

Aiming to solve the reliable and efficient distributed task assignment problem in WSNs, task-aware reliable and efficient task allocation for WSNs is investigated in this paper. The contributions of this paper are summarized as follows:

1) A global network view consisting of a multi-level view (including physical topology, routing topology, and task view) is constructed as a conceptual basis for global optimization;
2) A reliability evaluation model is established from the perspective of the task, and the constraint factors and objective functions of the task allocation are analyzed;
3) We propose the transmission-oriented reliable and energy-efficient task allocation (TRETA)-Cluster, in which the sink assigns reliability to all cluster heads according to the reliability requirements, and the cluster head performs local task assignment according to the assigned phase target reliability constraints. Simulation results show that TRETA-Cluster reduces the communication cost and latency of task assignment compared to centralized task allocation;
4) We propose the TRETA-Multi-Sink, where the global view is obtained by fetching local views from multiple sinks. Multiple sinks have a consistent comprehensive view of global optimization. The ways to respond to local task assignment requirements without the need to communicate with remote nodes overcomes the disadvantages of centralized task assignment in large-scale WSNs with significant communication overhead and delay, and have better scalability.

The remainder of this paper is structured as follows. Section 2 introduces the related work, the task allocation problem in WSNs is described in Section 3, and a global network view consisting of a multi-level view is proposed in Section 4. Next, we present a reliable and efficient task allocation algorithm for WSNs in Section 5, including centralized task allocation and two distributed task assignments. The performance is evaluated by theoretical analysis and simulation in Section 6, and finally, concluding remarks and future work are discussed in Section 7.

## 2. Related Work

Many research results on distributed task assignments for WSNs have been achieved, although most methods aim solely at maximizing the network lifetime [1,2]. Yu Wanli et al. propose an optimal online task assignment algorithm DOOTA (Distributed Optimal On-line Task Allocation) that is suitable for multi-partition scheduling by taking into consideration the energy cost of communication, calculation, sensing, and sleep activities [1]. To solve the problem of task allocation on the Internet of Things-based applications, considering special functions and design features, Khalil Enan et al. proposed a new task group and a virtual object-based framework and then adopted a meta-heuristic solving method [2] by modeling the problem as a single-objective optimization problem with the primary goal of minimizing energy consumption.

In addition to considering the extension of the network lifetime, part of the work also takes into account the quality of service (QoS) requirements [3,4]. Using fuzzy inference systems, Ghebleh Reza et al. proposed a method for discovering multi-criteria resource discovery in the context of distributed systems [4]. The main disadvantage of this is that the computational overhead increases rapidly as more requests and resources are invoked. Several researchers have applied game theory to WSNs in order to solve task allocation problems [5,6]. To solve the selfish behavior of some nodes, some have adopted the cooperative enforcement games strategy [7]. Despite that, the QoS requirements they satisfy are mainly focused on minimizing completion time, load balancing, data sampling rate, and accuracy, among others. Among the requirements to be considered, there is no research work on reliability requirements. Alternatively, some researchers have addressed the problem of distributed task allocation in WSNs from cloud-based architectures [8,9]. Though, due to the distance between the cloud platform and the user, the group perception has higher waiting times.

Based on the self-organizing characteristics of WSNs, Ye et al. divided the implementation techniques of distributed task allocation into two types [10]: one is based on reinforcement learning [11], and another is a collaboration based on nodes—the local interaction between nodes in a collaborative manner to achieve a self-organizing task assignment. For example, based on the auction method [9,12] and the distributed task allocation based on the negotiation method [13], the two methods can achieve optimal results since they are obtained through negotiation between the two parties, which differs from other methods in that they use only a specific algorithm or a specific set of algorithms to obtain results. It happens that substantial communication overhead cannot be avoided during the negotiation process, so the auction-based and negotiation-based approaches are not suitable for resource-constrained WSNs.

The task assignment based on auction and negotiation has a significant communication load in a typical distributed task allocation due to the frequent interaction between nodes and unsuitable resource-limited WSNs. Moreover, the task allocation based on reinforcement learning also faces the problem of longer convergence time. In research that utilizes intelligent algorithms to solve the optimization goals of task assignment, such as particle swarm optimization, it is easy to fall into the optimal local problem. Wang et al. proposed a globally optimized task allocation algorithm based on an ant colony algorithm that requires a global pheromone matrix to obtain the optimal solution [14]. However, the authors missed mentioning how to construct a three-dimensional (3D) path pheromone storage space and the cost for the construction of this 3D space.

The research status presented above prompted the authors to investigate the distributed reliable task allocation problem in WSNs in order to develop an optimal global solution for reliable and efficient task allocation problems at a minimal cost. As is known, the transmission task is the most crucial issue in WSNs and thus the goal is to minimize the energy consumption of task allocation in WSNs based on the deadline and the reliability of tasks.

Two task allocation strategies under the constraints of reliability and task deadline are proposed in this paper, named TRETA-Cluster and TRETA-Multi-Sink. The term “task” represents the “transmission task” in the subsequent parts of this paper.

## 3. Description of Reliable and Efficient Task Allocation in WSNs

### 3.1. Energy Consumption Model

The total energy consumption of a task $m_{i}$ is the sum of the energy consumed on all nodes that undertook such a task. That is, the energy consumption of a node includes computational and communication energy consumption associated with that task. For calculation purposes, the energy model depicted in [15] is used in this paper, so $e_{ij}$ represents the energy consumed by the task $m_{i}$ in node $N_{j}$. When the node $N_{j}$ is not selected by the task $m_{i}$, $e_{ij}=0$. Otherwise, $e_{ij}$ is composed of the calculated energy consumption and communication energy consumption of node $N_{j}$. The calculated energy consumption of $N_{j}$, namely $e_{ij}^{comp}$, is given by Equation (1):

(1) $$e_{ij}^{comp}=e_{j}\times t_{ij}^{comp}$$

where $e_{j}$ represents the average processing energy consumption of the nodes in the network, and $t_{ij}^{comp}$ is the calculation time of task $m_{i}$.

The communication energy consumption of a node, namely $e_{ij}^{comm}$, includes the energy consumption for transmitting and receiving data packets. According to the commonly used communication energy model [6], the energy consumption of transmitting and receiving data of length $l_{ij}$ bit at the distance d is calculated by Equations (2) and (3):

(2) $$e_{ij}^{t}=\left(e_{elec}+\varepsilon_{amp}\times d^{2}\right)\times l_{ij}$$

(3) $$e_{ij}^{r}=e_{elec}\times l_{ij}$$

where $e_{elec}$ is the energy consumption of operating the radio model for each bit, and $\varepsilon_{amp}$ is the coefficient of the transmit amplifier. The communication energy consumption of the node is calculated by Equation (4):

(4) $$e_{ij}^{comm}=e_{ij}^{t}+e_{ij}^{r}$$

Thus, the energy consumption of task $m_{i}$ in node $N_{j}$ is then calculated using Equation (5).

(5) $$e_{ij}=e_{ij}^{comp}+e_{ij}^{comm}$$

### 3.2. Constraints of Task Reliability and Deadline

#### 3.2.1. Task Deadline Constraint

Let $P_{i}$, $S_{i}$ and $C_{i}$ represent the task period of $m_{i}$, the task start time and the task end time (deadline) respectively. The assignment of task $m_{i}$ thus needs to be satisfied with the deadline constraint:

(6) $$C_{i}\geq P_{i}+S_{i}$$

#### 3.2.2. Task Reliability Constraint

As is known, a WSN consists of a large number of randomly distributed sensor nodes. Performing different tasks in the same network will also show different task reliability due to factors such as network traffic and transmission path; thus, a scheme that matches the task assignment of WSNs with task reliability requirements becomes necessary. Task assignment needs to satisfy Equation (7):

(7) $$R_{WSN}\geq R_{s}$$

where $R_{WSN}$ represents the transmission reliability of the current WSN, whose value can be obtained from the evaluation model of reliable transmission given in Equations (8) or (10). Also, $R_{s}$ represents a threshold for the application of WSN transmission reliability.

Reliability is an important indicator to measure the QoS of WSNs. It has been well studied in the past and is mainly regulated by the successful delivery rate of the link packets. Users are more concerned with the quality of the transmission of a task than the quality of the link in many applications [16]. Since a task is contained by many packets, the user is concerned whether the end-to-end event is successfully perceived rather than the successful delivery rate of the individual node’s data packets. Therefore, it is necessary to measure the reliability of WSNs from the perspective of transmission and how the tasks in the network are utilized based on the analysis of the granularity.

For two typical topologies in WSNs, mesh (planar) and cluster-based (hierarchical), the cognitive data transmission process of these conventional topologies is analyzed. A task reliability evaluation model is then established based on the transmission path.

(A) Clustered Topology

Clustered topology is widely used in a variety of applications due to its higher energy efficiency and scalability. The heads of different clusters compose the backbone layer of WSNs. When evaluating a transmission task, it is supposed there are *L* task-related clusters in the sensing area. The reliability of clustered WSNs at time t is:

(8) $$ClusterR_{s_{i,}sink}\left(t,i\right)=\overset{L}{\underset{c=1}{\prod }}R_{A}^{C}\left(t,i\right)\times R_{T}^{c}\left(t,i\right)$$

where $R_{A}^{c}\left(t,i\right)$ refers to the reliability of the i-th transmission performed by cluster *c*. In most clustered WSNs, a cluster is designed with one cluster head, and the transmission between cluster head and members is single-hop [17]. Since the packet delivery rate is within one hop in a cluster, the error between the average and the actual value is smaller. Therefore, $R_{A}^{c}\left(t,i\right)$ can be modeled by a k-out-of-n system under the multisource environment.

Let $R_{T}^{c}\left(t,i\right)$ represent the reliability of the head of cluster *c*, which successfully sends the collected data of *i*-th transmission to sink at time *t*. This stage is considered successful as long as there is at least one path whose packet delivery rate is higher than the transmission threshold. Thus, the head of cluster *c* which successfully sends the collected data of the *i*-th transmission to sink at time *t* can be modeled by a parallel system.

(B) Mesh Topology

In a mesh WSN, there are multiple source nodes randomly located in the perceived area. The sink is the destination in the uplink transmission, as multiple source nodes collect data packets independently yet transmit them to the sink via intermediate nodes. The transmission reliability $MeshR_{s_{i},sink}\left(t,i\right)$ in mesh WSN is shown in Equation (9), where K is the number of source nodes, and $R_{s_{i},sink}\left(t,i\right)$ represents the reliability of the transmission task from the source $s_{i}$ to the sink in the network at time t.

(9) $$MeshR_{s_{i},sink}\left(t,i\right)=\overset{K}{\underset{s_{i}=1}{\sum }}R_{s_{i},sink}\left(t,i\right)$$

Let l represent the number of paths in the minimal path sets from the source $s_{i}$ to sink. At time t, $R_{s_{i},sink}^{i}\left(t,i\right)$ is the probability that there exists at least one path whose packet delivery rate is greater than the threshold of *i*-th transmission in the disjoint minimal path sets (from $s_{i}$ to sink) is shown in Equation (10).

(10) $$R_{s_{i},sink}^{i}\left(t,i\right)=1-\overset{l}{\underset{j=1}{\prod }}[1-R_{path\left(s_{i},sink\right)}^{j}\left(t,i\right)]$$

### 3.3. Reliable and Efficient Task Allocation Problem Model

WSNs can be represented by graph G(V,E). M(t) represents the set of tasks to be allocated at time t, and $m_{i}\in M\left(t\right)$ represents the *i*-th task in M(t). In a task allocation for WSNs, the two processes of task mapping and task scheduling are included. First, each task is mapped to the sensor node in the graph G(V,E) that is represented by the function $\varphi (m_{i}):M\to V$. When a task is assigned to multiple nodes, communication task scheduling is performed between the nodes, and the process of path allocation is represented by the function $\phi (m_{i}):L\to E.$ Therefore, the reliable and efficient WSNs task allocation problem can be abstracted into the following constraint optimization problems:

Input:      TG(V,E), RT(S, Ps), M(t)
Output:    $\varphi (m_{i}):M\to V$, $\phi (m_{i}):L\to E$

Satisfying Equation (11):

(11) $$\begin{array}{l} \min\;\;Eng\varphi (m_{i})=\sum_{\tau \in V}e_{ij},\;\;\;m_{i}\in M\left(t\right)\\ s.t\;\;R_{WSN}\geq R_{s},\;\forall \tau \in V\\ \;\;\;\;\;\;C_{i}\geq P_{i}+S_{i} \end{array}$$

where TG(V,E) represents the physical topology graph of the network, V represents the set of sensor nodes in the network, E represents the set of links between the nodes in the network, RT(S,Ps) represents the routing topology of the network,S represents the source node set and Ps represents the path set of all source nodes to the sink in the network. $Eng(\varphi (m_{i}))$ is the energy consumption of task $m_{i}$ that is equal to the sum of the energy consumption on all nodes in the network assigned to task $m_{i}$. From this, Equation (11) indicates that the goal of the task allocation to WSNs is to minimize energy consumption under the constraints of reliability and deadline. Given that this is a nonlinear mixed-integer programming problem, it can be solved using a heuristic algorithm.

## 4. Global Network View

The physical topology of a WSN, the routing topology, and the set of tasks to be allocated at time *t* are known as input data assigned by the task in Equation (11). Additionally, the sensor nodes in a WSN learn local topologies through topology discovery, which are periodically sent to sink nodes to form the global physical topology of the network.

### 4.1. Route Topology

**Figure 1:**
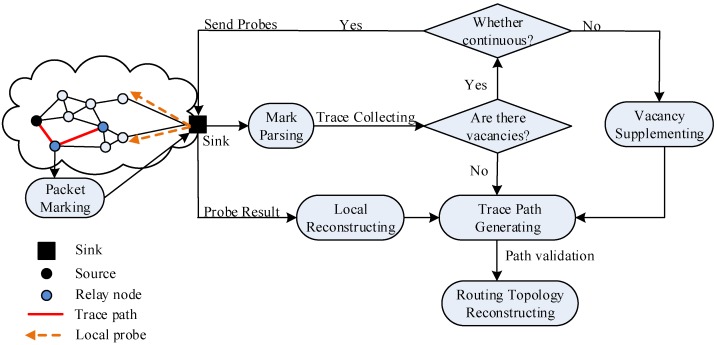
The overall framework of route topology interference (RTI).

We proposed the route topology inference (RTI) in [18]. The algorithm framework is shown in Figure 1. Since this method is not limited by the routing protocol adopted by the current network, and only uses the packet tracking hybrid active detection method to construct a transmission path from the source node to the sink, an online global routing topology view for a WSN can be provided and shown.

The core idea of packet tracing is that, on the transmission path from the source node to sink, the forwarding nodes selectively mark part packets according to the marking rule upon receiving the packets. At the end of the sampling period, the sink can establish trace lists from the source node to the sink by parsing the marked packets. As the sink determines the one-to-one correspondence trace in the trace list according to the packet with the same hop to the source node, it retrieves the trace list from the source node. The trace list returned by mark parsing may be incomplete due to packet loss or insufficiently marked packets. Also, the vacant traces in the trace list can be supplemented by auxiliary inference or active detection [18].

RTI increases the memory load, though it has a significant advantage in the correctness and convergence of reconstruction. Moreover, the relay node does not need to mark all packets, as it only marks the packets based on conditions, and for one path, each packet is marked only by one relay node.

The reconstructed WSN routing topology is shown as a graph, where the nodes on each path and links between the nodes are added to the graph, and the source node identifying a path is added to the link also. If a source node has multiple paths to the sink, the remaining nodes or edges are added to the routing topology based on the tracking path after the first path is added, so the marked routing topology view from the source node to the sink is generated.

### 4.2. Multi-Level Global View

**Figure 2:**
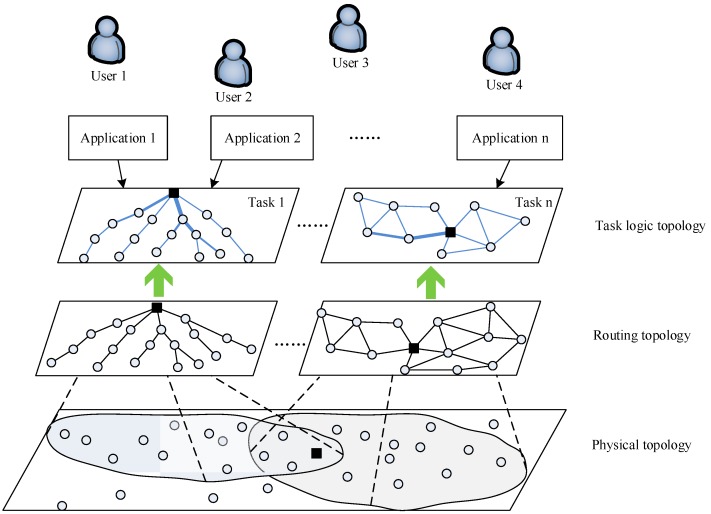
The multi-level global view of a wireless sensor network (WSN).

Once the routing topology is acquired, the task logical topology of different tasks in the current network is further abstracted according to the task mark in the data packet. As shown in Figure 2, the architecture that provides a conceptual basis for global optimization of network management and the multi-level global view architecture consisting of the physical topology, the routing topology, and the task logical topology is obtained. The routing topology layer can generate different routing topologies for applications according to different node sets in the physical topology layer (i.e., different shadows in Figure 2). Also, links of different thicknesses in the task logic topology reflect the current traffic of the link.

## 5. Reliable and Efficient Task Allocation Algorithm for WSNs

### 5.1. Centralized Task Assignment

As presented before, the aims at this research are to minimize the energy consumption of task allocation under the constraints of reliability and deadlines in the task allocation model problem presented in Section 3, where TG(V,E), RT(S,Ps) and M(t) are the three inputs as presented in Equation (11).

TRETA is performed by the sink based on the given task reliability and deadline constraints through a global view, and its framework consists of five modules, enumerated as: data collection, task reliability evaluation, topology management, event management, and task assignment. The data collection module collects state information of each node, such as node ID (Identity Document ), node residual energy information, node neighbor, and packet loss rate information, among others; the node collection module stores network state information such as nodes, paths and task information (transmitted from the application layer) in the corresponding node table, topology information table, state information table, and task information table in the database for extraction by other modules; and the topology information table in the data collection module stores the global physical topology of the network.

To establish a global view of the current network state, a topology management module in the framework of TRETA is designed. In addition to the global physical topology, the topology management module obtains the routing topology of the global network and then constructs a two-level global view.

The event module manages events and triggers other module updates when a drive event occurs. To achieve reliable and efficient task allocation in WSNs, the driver events concerned include topology change (nodes join or leave), as the reliability of the task is lower than the threshold and new tasks to be allocated.

**Figure 3:**
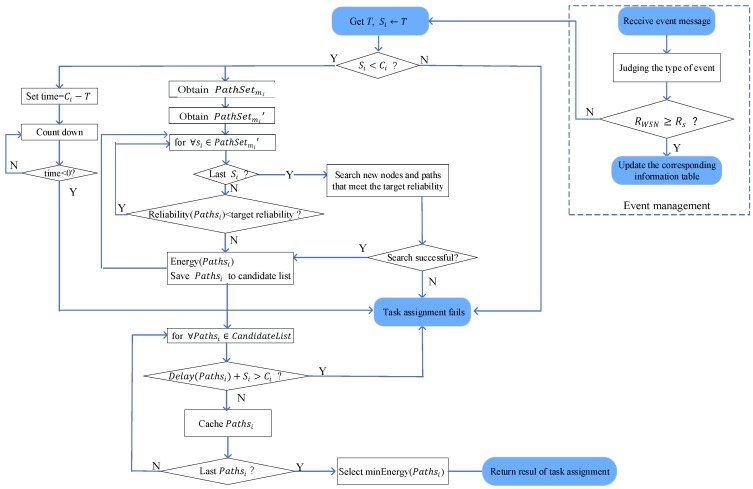
Transmission-oriented reliable and energy-efficient task allocation (TRETA) algorithm.

Based on the modules presented above, a reliable and efficient task allocation algorithm, namely TRETA, is proposed, as depicted in Figure 3.

During the execution of the network and once the defined driving events occur, the event management module triggers the update of the data collection module, and the sink determines whether the reliability of the updated network is less than the target reliability. If yes, the task allocation module is notified to re-assign; otherwise, only the global view corresponding to the event is updated.

### 5.2. Distributed Task Assignment

Since TRETA performs task assignments from the perspective of global optimization, it has the disadvantage of significant communication overhead and delays in large-scale WSN. Aiming to obtain reliable and efficient distributed task allocation in WSNs, two distributed task assignments for WSNs based on TRETA are proposed.

#### 5.2.1. Distributed Reliable and Efficient Task Allocation in a Hierarchical Topology

**Figure 4:**
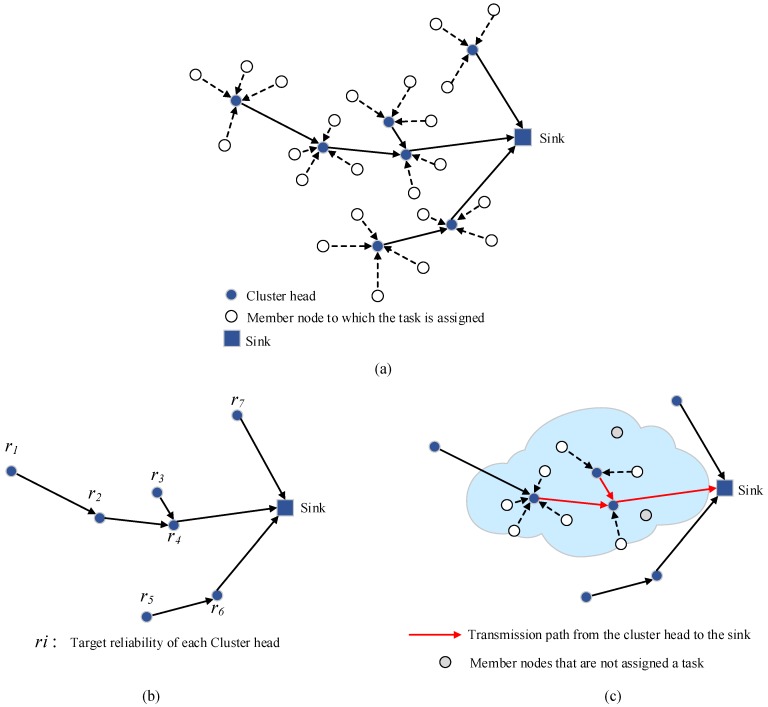
Reliable and efficient distributed task allocation based on clustering: (**a**) Cluster-based WSNs; (**b**) Sink assigns reliability to the cluster head; (**c**) Cluster head performs local task allocation.

A hierarchical topological diagram of a clustered WSN, where the sink is the center of the entire network, is depicted in Figure 4a. The cluster head saves the collected local physical routing topology as well the state information of the member nodes in the local cluster, passing them next to the sink. Then, the sink node can obtain the physical topology of the entire network by merging all the topologies received, so the topology management module receives a two-level global view.

Based on the above observations, we present a distributed reliable and efficient task assignment algorithm based on TRETA for clustered WSNs, named TRETA-Cluster, which divides the task allocation into two phases: backbone network and intra-cluster allocation.

1) The sink performs the reliability distribution at the backbone network composed of the cluster heads according to the target reliability, where reliability $r_{i}$ is obtained at each cluster head, as shown in Figure 4b. Combined with the target reliability and the task deadline constraint, the sink calls the TRETA algorithm to select the cluster head with the smallest total energy consumption that satisfies the task deadline and the target reliability constraint, and the transmission path of the cluster head to the sink.
2) The cluster head calls the TRETA to select the nodes in the cluster and the intra-cluster paths according to the obtained intra-cluster reliability index and local view to realize the task allocation of the cluster.

If the task allocation is successful, the allocation result is returned; otherwise, the cluster head selects the node that satisfies the target reliability according to the local physical topology. In case the node is found, the allocation result is returned. The task allocation fails and returns if otherwise, as shown in Figure 4c.

As a topology change occurs, the reliability may become lower than the threshold or the cluster head can be interrupted. In this case, the task allocation is re-executed by the sink or the cluster head according to the rule, as seen in Algorithm 1, TRETA-Cluster, Lines 17–20.

The reliability allocation [19,20] is to assign the target reliability of a task to the appropriate subsystems, components, and nodes of the system to determine the reliability of each component. Referring to the reliability allocation algorithm in [21], we assign the target reliability of the task to the two stages and use them as the target reliability of each stage (Algorithm 1, TRETA-Cluster, Lines 5–6; RA is the reliability distribution function).

**Algorithm 1.** TRETA-ClusterInput: $TG\left(V,E\right),\;RT\left(S,Ps\right),\;M\left(t\right),\;R_{s},\;C_{i}$ Output: $\varphi (m_{i}):M\to V,\;\phi (m_{i}):L\to E$
1 for $\forall m_{i}$, $m_{i}\in M\left(t\right)$2   get T, $S_{i}=T$3   if $S_{i}<C_{i}$4   for ∀CH, CH∈RT(S,Ps) //CH represents the cluster head5     $R_{CH}=RA\left(RT\left(S,Ps\right)\right)$6     $R_{CM}=RA\left(RT\left(S,Ps\right)\right)$7   for $\forall CH\in RT_{b}\left(S,Ps\right)$8     call TRETA $\left(TG_{b}\left(S,Ps\right),RT_{b}\left(S,Ps\right),R_{CH}\right)$9   for ∀ Cluster∈RT(S,Ps)10     call TRETA $\left(TG_{c}\left(S,Ps\right),RT_{c}\left(S,Ps\right),R_{CM}\right)$11     if call TRETA $\left(TG_{c}\left(S,Ps\right),RT_{c}\left(S,Ps\right),R_{CM}\right)\neq \emptyset$12     return allocation result13  get T14  if $T>C_{i}$, return line 115  if CH fault16     sink update TG(V,E) and RT(S,Ps)17     if $R_{WSN}<R_{s}$, then18      call TRETA $\left(TG_{b^{\prime }}\left(V,E\right),\;RT_{b^{\prime }}\left(S,Ps\right),R_{CH^{\prime }}\right)$19      elseif the failed cluster is re-clustered20      sink update TG(V,E) and RT(S,Ps)22  if member of CH change23      sink update TG(V,E) and RT(S,Ps)24      if $R_{WSN}\geq R_{s}$, then return25      else26      call TRETA ($TG_{c^{\prime }}\left(V,E\right),RT_{c^{\prime }}\left(S,Ps\right),R_{CM})$27      if call TRETA ($TG_{c^{\prime }}\left(V,E\right),RT_{c^{\prime }}\left(S,Ps\right),R_{CM})\neq \emptyset$28      return allocation result29      else return line 130 if $R_{WSN}<R_{s}$, then31   return line 132 return


It can be noted that the local task allocation of the cluster head may select the node participating in the task, and since the path from the cluster head to the sink has been obtained in the first stage, the selection from the node to the path is completed in two phases in a task assignment.

Whenever the nodes in the cluster change (join or leave), the cluster head transmits the topology change to the sink. The sink determines whether the node change affects the reliability of the task. If not, it returns. Otherwise, the cluster head node tries to re-select the node in the cluster after the change. Additionally, if the intra-cluster target reliability constraint can be met, the cluster head re-performs the local task assignment. Otherwise, the sink performs the task allocation again.

When the sink finds that the task transmission is below the reliability threshold, the task allocation is performed again. However, when the cluster head fails, the cluster will disconnect from other parts of the network and the task assignment policy issued by the sink cannot reach the cluster, bringing challenges to the reliable task allocation of WSNs. The proposed processing scheme follows: the sink updates the two-level global view and determines whether the fault of the cluster has an impact on the target reliability of the current task. If so, the task is reassigned according to the updated two-level global view. Otherwise, the sink only updates the two-level global view that reduces the frequency of the update. After the faulty cluster re-selects the cluster head, the cluster head collects the cluster topology and the sink node updates the global view and reperforms the task assignment.

#### 5.2.2. Distributed Reliable and Efficient Task Allocation in Planar Topology

For a large-scale WSN, perceptual information needs to go through multi-hop communication to the sink. Considering the overhead of establishment, maintenance routing by node, and long-distance multi-hop communication delay in planar topology to ensure the reliability and scalability of WSNs, some researchers have proposed the multi-sink deployment scheme [22,23,24,25] or software-defined network (SDN) controller [26].

**Figure 5:**
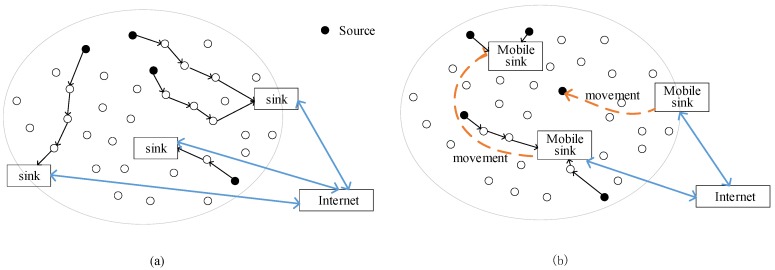
Multi-sink deployed WSNs. (**a**) Static multi-sink WSNs; (**b**) mobile multi-sink WSNs.

The deployment strategies of multiple sinks in a planar topology include static and dynamic, random, and scheduled deployment methods. As an illustration, the static and mobile deployment of multiple sinks is shown in Figure 5.

In a WSN with multiple sinks deployed, this section proposes a distributed task assignment strategy based on multiple sinks. Multiple sinks in this strategy have a global view of the entire network, enabling local requirements to be globally optimized and without the need to communicate with remote nodes, reducing the communication cost and latency of task assignments.

(A) Consistency of Multi-Sink Global View

Due to the geographical distribution and asynchronous operation features, when the sink publishes the global view, the update time of multiple sinks is not synchronized due to network delay or other reasons, resulting in inconsistency in the forwarding or processing of the task data. To share the global view among multiple sinks, it is necessary to consider the consistency problem of the global views between multiple sinks. In a resource-constrained WSN, how to efficiently share global view among multiple sinks to achieve fast and efficient global task allocation is still one of the significant questions.

In Eric Brewer’s consistency, availability and partition tolerance (CAP) theory, it has been proved that consistency, availability, and partition tolerance in a distributed system cannot be considered together [27]. Partition occurs easily in WSNs. If strong consistency is guaranteed, the availability of the network cannot be guaranteed at the same time, and the communication cost of realizing the strong consistency of multiple sink global views in large-scale WSNs is high, the final consistency is chosen to reduce the communication cost of synchronization between nodes.

In an existing final consistency technology, Dynamo, the storage platform of the key-value pattern of Amazon has been paid more attention by many researchers [28,29]. Dynamo proposes an NWR model that guarantees eventual consistency, where N represents the number of copies of data being saved, R represents the number of copies required for each read success, and W represents the number of copies as are necessary for each write success. By setting R and W, when R+W>N, it produces a system similar to Quorum.

Quorum is widely used in distributed storage systems [30,31,32,33]. It is a set and a subset of all copies C, where two parameters W and R are pre-defined for N copies, and N=|C|, W≥1, R≤N. The quorum set of writing is as follows:

(12) $$S_{W}=\left\{Q|Q\subseteq C\wedge \left|Q\right|=W\right\}$$

and the quorum set of reading as:

(13) $$S_{R}=\left\{Q|Q\subseteq C\wedge \left|Q\right|=R\right\}$$

If R+W>N, there is an intersection between $S_{W}$ and $S_{R}$, so any read operation can return the latest write and guarantee strong consistency. Conversely, if R+W≤N, then the two elements overlap with probability $P_{overlap}$ [29], as in Equation (14):

(14) $$\begin{array}{ll} P_{overlap} & =1-\frac{\left(\begin{array}{c} N\\ W \end{array}\right)\left(\begin{array}{c} N-W\\ R \end{array}\right)}{\left(\begin{array}{c} N\\ W \end{array}\right)\left(\begin{array}{c} N\\ R \end{array}\right)}\\ & =1-\frac{\left(\begin{array}{c} N-W\\ R \end{array}\right)}{\left(\begin{array}{c} N\\ R \end{array}\right)} \end{array}$$

For a write request, it is first sent to the replica as a coordinator, and then the coordinator propagates the write to all other replicas. After getting at least W−1 responses, the coordinator returns. Read requests are handled in the same way as write requests, although the coordinator should wait for at least R−1 responses. If the read replica set overlaps with the write replica set, the read request can return the most recent value. Otherwise, it will return stale data.

Similarly, this paper proposes a method to ensure the final consistency of multiple global views. In this method, each sink maintains a log in addition to the global view that includes the version of the current global view (new), the version of the previous global view (old), and the update operation from the old version to a new version.

In a multi-sink WSN, if the network status changes, the changed local sink sends an update request to other sinks. If at least W−1 sinks return a response, the update request is considered successful, and the sink submits an update and broadcasts the update to other sinks. Next, the previously responded W−1 sinks update the current global view version, the previous version of the global view, and the update operation from the old version to the new version in the log. As soon as a sink node initiates a task assignment, the global view needs to be read. In addition to reading the locally saved global view, the sink node issues a read request to the other sinks, then waits for R−1 responses, and finally returns the read result.

Since the task allocation has a deadline constraint, to reduce the delay of reading the global view, the algorithm sets R=2 to read the global view of two sinks. Therefore, the complexity of guaranteeing consistency is pushed to the write operation; that is, for the network state update process which is not sensitive to delay, as long as R+W>N is satisfied, the final consistency can be guaranteed. If concurrent partial updates occur in the network at the same time, the concurrency control protocol is used for processing.

(B) Task Assignment Based on Multi-Sink WSNs

In a planar WSN topology where multiple sinks important, these sinks form a multicast group, and the multiple sinks are equal. To simplify the design, it is assumed that multiple sinks can remain fully connected and the sinks do not fail, as each sink collects the local view, including the physical topology and routing topology. The local views are periodically exchanged so that multiple sinks in the multicast group gradually obtain a global view of the network.

The concept of TRETA-Multi-Sink follows: after each sink has acquired the global view and there is a task to be allocated, if the current time of the system is less than the deadline of the task to be assigned, the sink closest to the task can be selected to call the TRETA algorithm for local optimization allocation. Since the sink has a global view and despite a local allocation, the sink can perform a globally optimized task assignment. In the algorithm, multiple sinks select the final consistency when exchanging global views to reduce the communication cost of synchronization between sinks. If the current time of the system is higher than the deadline of the task to be assigned, the current task allocation is interrupted.

Specifically, for the task $m_{i}$ to be assigned, if the current time of the system is less than the deadline of the task, the nearest sink of task $m_{i}$ is found through the physical topology information and is represented by $sink_{j}$, which reads the local and adjacent global view version. To ensure the final consistency of the global view between multiple sinks, the reading number of copies is R according to the NWR model (we let R=2); if the versions are different, it will read the global view with the latest version. Otherwise, it will read the local - global view of $sink_{j}$. Then, $sink_{j}$ calls the TRETA algorithm for local optimization allocation. During this process, if the current time of the system exceeds the task deadline, the task allocation is interrupted.

When the network update occurs, if the scope of the update involves only one sink (represented by $sink_{k}$), then $sink_{k}$ multicasts the update request to other sinks. According to the NWR model, the number of copies that need to be written to the update is W−1. W in this paper should satisfy W>N−2.

If the number of sinks in response to the update request is higher than W−1, $sink_{k}$ submits the view update, and other sinks in the multicast group update the local-global view accordingly. Otherwise, the update of $sink_{k}$ is deleted to avoid inconsistency with other different sink views. 

When the sink finds that the task transmission is lower than the reliability threshold, the task allocation is re-executed. The specific process of Algorithm 2, TRETA-Multi-Sink is as follows:
**Algorithm 2.** TRETA -Multi-sinkInput: $TG_{j}\left(V,E\right),\;RT_{j}\left(S,Ps\right),\;M\left(t\right),\;R_{s},\;C_{i}$Output: $\varphi (m_{i}):M\to V,\;\phi (m_{i}):L\to E$1 $TG\left(V,E\right)=\mathrm{Merge}(TG_{j}\left(V,E\right)),\;j=1,2,\ldots n$2 $RT\left(V,E\right)=\mathrm{Merge}(RT_{j}\left(V,E\right)),\;j=1,2,\ldots n$3 for $\forall m_{i}$, $m_{i}\in M\left(t\right)$4    get T, $S_{i}=T$5    if $S_{i}<C_{i}$6    $sink_{j}=\left\{sink|\mathrm{Mindistinct}\left(m_{i},sinks\right),sink\in TG_{j}\left(V,E\right)\right\}$7    read $sink_{j}.viewID\;\mathrm{and}\;sink_{jn}.viewID$8    if $sink_{j}.viewID\neq sink_{jn}.viewID$9      read view of $\mathrm{latest}\left(sink_{j}.viewID,sink_{jn}.viewID\right)$10   else read $sink_{j}.RT_{j}\left(S,Ps\right)$11   call TRETA ($TG_{j}\left(V,E\right),RT_{j}\left(S,Ps\right),M\left(t\right),\;R_{s})$12   get *T*13   if $T>C_{i}$, return line 114   if TG(V,E) change happens and Num(changed sink)=115    $sink_{k}$ record change in cache16    $sink_{k}$ multicast update-request to other sinks17      if $Num\left(sink_{k}^{res}\right)>W-1$18        $sink_{k}$ submit an update19        $\mathrm{update}\left(sink_{k}.TG_{j}\left(V,E\right),\;sink_{k}.RT_{j}\left(S,Ps\right),sink_{k}.log\right)$20        $\mathrm{update}\left(sink_{k}^{res}.TG_{j}\left(V,E\right),\;sink_{k}^{res}.RT_{j}\left(S,Ps\right),sink_{k}^{res}.log\right)$21      else delete $sink_{k}.record$22   if $R_{WSN}<R_{s}$, then23     return line 324 return

## 6. Performance Analysis

### 6.1. Theoretical Analysis

In this section, the performance of distributed reliable and efficient task allocation is analyzed primarily from the algorithm complexity, communication load, and delay of the task assignment.

#### 6.1.1. Algorithm Complexity

TRETA-Cluster is divided into two phases. In the first phase, the sink performs task allocation for the backbone network composed of the cluster heads and the sink, with the worst-case calculation complexity $O\left({\left(\alpha N\right)}^{3}\right)$, where α represents the ratio of the size of the backbone network to the size of the entire network system. During the second phase, the cluster head is responsible for the task allocation within the cluster. The calculation complexity of this process is $O\left({\left(\gamma N\right)}^{3}\right)$, where γ represents the ratio of the cluster member nodes size to the entire network size. Based on the above process, the calculation complexity of distributed task allocation based on clustering topology is $O\left(N^{3}\right)$.

In TRETA-Multi-Sink, the calculation complexity of multiple sinks in local task allocation is the same as TRETA, although the processing of global view consistency is added to the network update in TRETA-Multi-Sink, which includes the write operation of multiple sinks when global view update occurs and read operation when task assignment occurs. Assuming N is the network size, β is the ratio of the part of the network to be updated, and W is the number of global views that need to be written, the calculation complexity of the write operation is O(βWN). Let the number of global views that need to be read is R (R=2); then, the calculation complexity of the read operation is O(2N). Based on the above process, the calculation complexity of the distributed task allocation algorithm is $O(N^{3})$.

The two distributed task allocation algorithms above reduce the size of the network. As noted, the distributed task allocation has the polynomial computation complexity in the background of global optimization.

#### 6.1.2. Communication Load of the Distributed Task Assigned

In the distributed task allocation strategy for the clustered topology, the sink multicasts the allocation policy to the selected cluster heads that are responsible for the task allocation within the cluster. Therefore, the communication load depends on the amount of information in the sink’s allocation policy. In this paper, the communication load of TRETA-Cluster is measured by the number of packets in the allocation policy by multicast, as shown in Equation (15):

(15) $$CM=Num_{cp}$$

For the multi-sink topology, the communication load includes: (1) multi-casting the global view between the sinks, namely $Num_{gv}$, where $Num_{gv}$ represents the communication load for transmitting one global view, and a represents the number of times of global views transmitted; (2) ensuring global view consistency between multiple sinks, i.e., the write load WM at update time and the communication load RW at read time, as shown in Equation (16):

(16) $$DM=aNum_{gv}+WM+RW$$

where only the version and update operation of the global view is transferred when the update is written, and performed only when the network is updated, as shown in Equation (17):

(17) $$WM=\{\begin{array}{l} VUM\\ 0 \end{array}$$

where VUM indicates the write load of the version and update operation of the global view when it is updated, and 0 means no update.

When the sink reads the global view, it needs to read two copies of the global view; that is, the global view of the local and that of the nearest sink in the network, as required by the final consistency. If the local version of the global view is newer, there is no need to communicate with other sinks, and thus the communication load of the read is 0. Otherwise, one global view needs to be transmitted. Therefore, the communication load RW when reading is as shown in Equation (18):

(18) $$RM=\{\begin{array}{l} 0\\ Num_{gv} \end{array}$$

#### 6.1.3. Delay of Distributed Task Assignment

In order to implement the task assignment, two known premises are required: the physical topology of the entire network and the routing topology of the network established in advance. They are considered in the initialization process for the task assignment, so the delay is not considered at initialization.

The task assignment delay for a clustered topology is calculated by Equation (19):

(19) $$RT_{d\_c}=RT_{c}^{\prime }+MC+CHD$$

where $RT_{d\_c}$ represents the delay of task assignment for the clustered topology, $RT_{c}^{\prime }$ represents the delay of the sink allocating a task to the subgraph composed of the cluster heads, MC represents the delay of the multicast performed by the sink, and *CHD* represents the delay in the allocation of the task by the selected cluster head.

Assuming that each sink has obtained a global view in a multi-sink deployment environment, this stage can be implemented by periodically swapping local views with each other through multiple sinks, requiring it as initialization prior to the task assignment. The delay for distributed task allocation based on multiple sinks is calculated by Equation (20):

(20) $$RT_{d\_d}=\bar{RT_{c}^{\prime \prime }}+C_{rt}.$$

where $RT_{d\_d}$ represents the delay of TRETA-Multi-Sink, $\bar{RT_{c}^{\prime \prime }}$ represents the average of the delays of the tasks assigned by multiple sinks locally, and $C_{rt}$ indicates the time to ensure the final consistency of the view, as shown in Equation (21):

(21) $$C_{rt}=\{\begin{array}{l} W_{rt}\\ R_{rt} \end{array}$$

where $W_{rt}$ indicates the time of writing of the global view whenever it is updated and $R_{rt}$ indicates the time of reading of the global view when the distributed task is assigned.

#### 6.1.4. Energy Resilience of the Transmission in WSNs

The nodes can go down for various reasons, e.g., the time of life or for the specific protocol used. In order to evaluate the energy resilience of the transmission in WSNs, we quoted an index η [34] as shown in Equation (22), where $N_{p}$ indicates the number of packets received by the sink after a fixed time, S indicates the number of initial active nodes, and D indicates the number of dead nodes after a fixed time.

(22) $$\eta =\frac{N_{p}}{S-D}$$

The index gives an indication of how efficient the network is in allowing information to be delivered considering both the number of packets that are running in the network and the number of nodes that are going out over time.

### 6.2. Simulation

#### 6.2.1. Simulation Design

In order to carry out a reliable and efficient task assignment, two distributed task assignment strategies are proposed, namely TRETA-Cluster and TRETA-Multi-Sink. In this section, a simulation is carried out using TOSSIM (TinyOS Simulator). In order to evaluate energy consumption, this paper expands TOSSIM and adds a power consumption model. The performance of the strategies is analyzed and compared in three aspects: different network size, task arrival rate, and network update rate.

In order to compare with the distributed task allocation, simulation of the centralized task allocation TRETA is also performed in the same network environment. The performance metrics analyzed include the energy allocated, the delay of successful allocation during the deadline, and the success rate of the task assignment. In addition, the simulation is carried out under the cluster topology and the multi-sink-based topology for the distributed task assignment.

1) Cluster topology
  The sensing area is 100 m × 100 m, and the number of sensor nodes is 50 to 300. There is only one sink in the cluster topology, with the sink node located in the center of the sensing area and remaining nodes randomly deployed.
2) Multi-sink-based topology
  The deployment strategies of multiple sinks in a multi-sink-based topology include static and dynamic, random, and scheduled deployment methods. In the proposed simulation experiment, the static uniform deployment method is selected, with the number of sinks set to five, and the sensing area of the square is evenly divided into four sub-areas. One of the five sinks is located in the center of the sensing area, while the remaining four sinks are located in the center of the respective remaining four sub-areas. The five sinks remain fully connected, and the case where the sinks fail is not considered.

The main simulation parameters are shown in Table 1. Three of the abbreviations ($e_{elec}$, $\varepsilon_{amp}$, and $e_{j}$) represent the energy consumption of operating the radio model for each bit, the coefficient of the transmit amplifier, and the average calculated the energy consumption of the nodes in the network, respectively. Kenneth et al. mentioned that wireless communication is usually the most energy-consuming process in traditional WSN applications [16]. Specifically, the energy required for a single bit transmission is 1000 times the energy consumed by calculating a single bit in classic 32-bit architecture, and thus the value of $e_{j}$ is set to 0.05 nJ/b.

The parameters of the task to be assigned are shown in Table 2, and three aspects are included: task type, transmission parameters, and task environment. Each simulation is executed for 120 minutes and repeated 500 times. For each performance index, we used the Monte Carlo method to obtain simulation results. The specific process is as follows: first, in the TOSSIM simulation environment, Python is used to generate a random number of each task parameter in the value interval based on a Poisson distribution. Secondly, each random variable is directly sampled, and simulation experiments and calculations are performed according to the task assignment strategy, and the optimal strategy of task assignment is obtained. Finally, statistical analysis is performed on the test results to obtain the average value of each evaluation index of the task assignment strategy.

#### 6.2.2. Simulation Results and Analysis

The performance evaluation indicators in this research include the energy consumption of task assignment, the delay of successful allocation within the deadline, and the success rate of the task assignment. The success rate of task assignment represents the ratio of the number of tasks successfully assigned to the total number of tasks to be assigned in a simulation cycle, while the other two indicators are calculated according to Equations (5), (19), and (20). For comparison purposes, the simulation of centralized task allocation in the same network environment is conducted and represented by TRETA.

(A) Energy Consumption for Task Assignment

**Figure 6:**
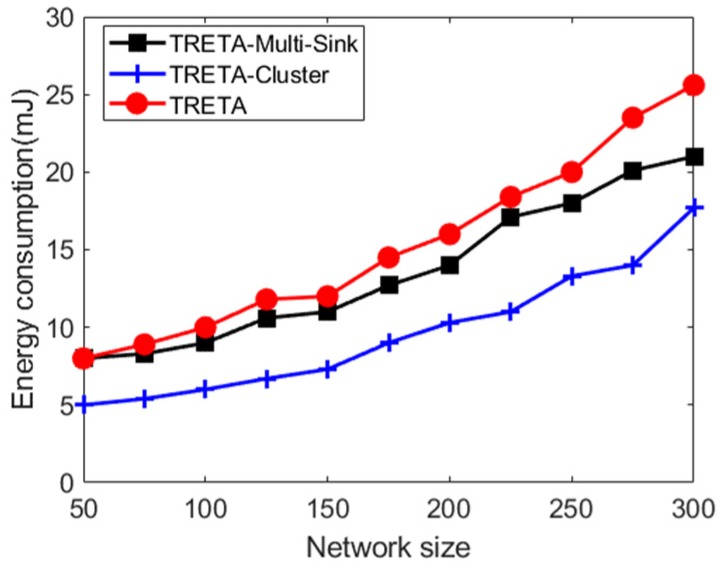
Distributed task assignment energy consumption.

It is shown in Figure 6, the comparison of energy consumption among the three allocation strategies is proposed: the centralized task allocation TRETA and the distributed task assignment strategies TRETA-Cluster and TRETA-Multi-Sink. As seen in this figure, the energy consumption increases with the increase of node size. Among them, the energy consumption of TRETA is highest, since the global state of the network is converged to a single sink through long-distance multi-hop in large-scale WSNs. On the other hand, the energy consumption of TRETA-Multi-Sink is relatively small, as it is based on the multi-sink being able to collect the local state and merge it into the global view by multicasting. TRETA-Cluster is performed in two stages. When the task cannot meet the target reliability, if the reselected node in the cluster can reach the stage target reliability, the sink does not need to re-allocate the task, so the energy consumption of TRETA-Cluster is less than both TRETA and TRETA-Multi-Sink.

(B) Delay of Successful Assignment within the Deadline

**Figure 7:**
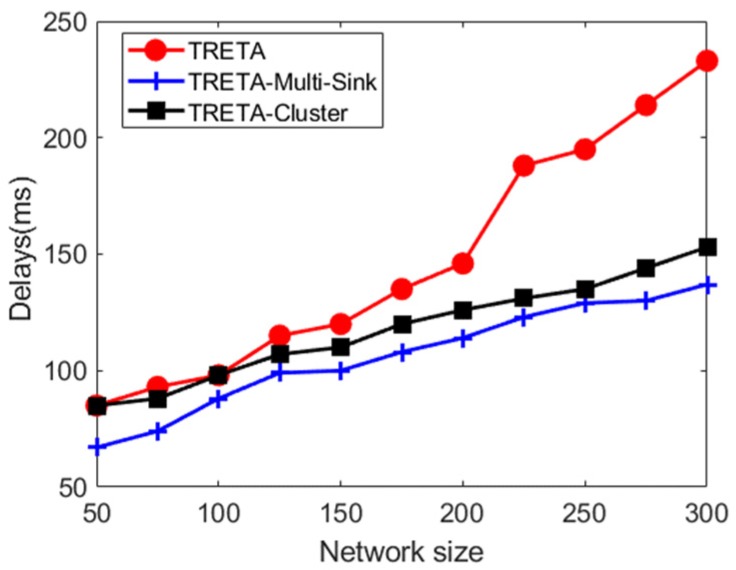
Task allocation delay.

The delay of task assignment in the size of the network from 50 to 300 nodes is analyzed. As can be seen in Figure 7, TRETA has a substantial delay. Due to long-distance multi-hop aggregation to a single sink, achieving the global state of the cluster topology causes considerable delays in large-scale WSNs.

(C) The Success Rate of Task Assignment

**Figure 8:**
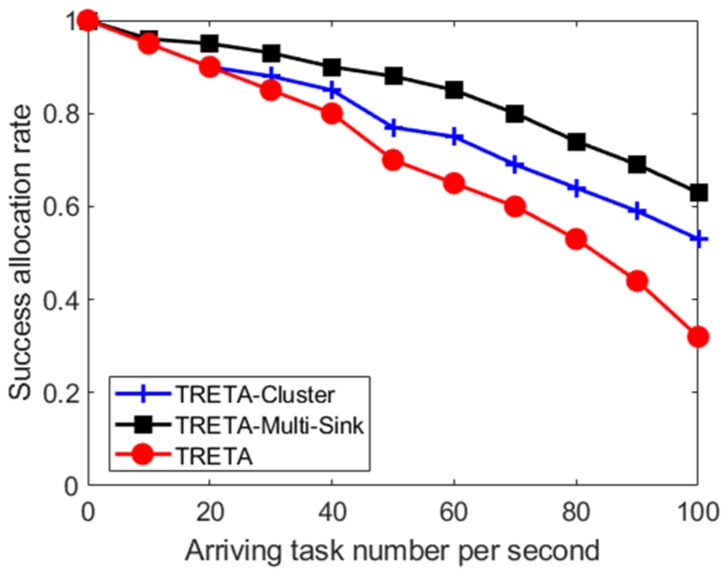
The success rate of task assignment under different task arrival rates.

The success rate of task assignments under different task arrival rates is analyzed. Such a rate represents the number of tasks waiting to be allocated per second, and the success rate of the task allocation represents the ratio between the number of successfully assigned tasks in one simulation cycle and the total number of tasks to be assigned. As seen in Figure 8, the success rate of the task allocation decreases as the task arrival rate increases, and the rise in the task arrival rate leads to the increase in link conflict in the network. Since the TRETA-Multi-Sink can be distributed to multiple sinks for local processing according to the task area, its success rate in the task assignment is better than TRETA and TRETA-Cluster. The higher the task arrival rate, the more prominent the advantage.

(D) Energy Consumption and Delay of Task Assignments in the Dynamic Update of the Network

**Figure 9:**
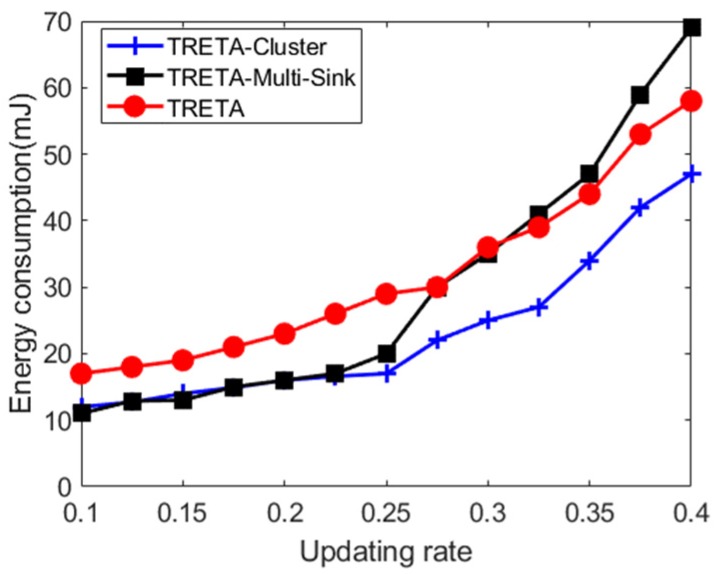
Energy consumption of task assignment under different network update rates.

Figure 9 shows the energy consumption of the three proposed strategies for different network update ratios. It is noted that as the network update ratio increases, the energy consumption is significantly increased. Since the network update in TRETA is brought to the only sink through the long-distance multi-hop, the communication load of the network is increased. Moreover, the energy consumption of TRETA is higher than the other two strategies in most cases. With the network update ratio greater than 25%, the energy consumption of TRETA-Multi-Sink increases rapidly, surpassing TRETA and TRETA-Cluster, becoming the highest among the three strategies due to the exchange of global views between multiple sinks and the operation of ensuring global view consistency under a high network update rate.

**Figure 10:**
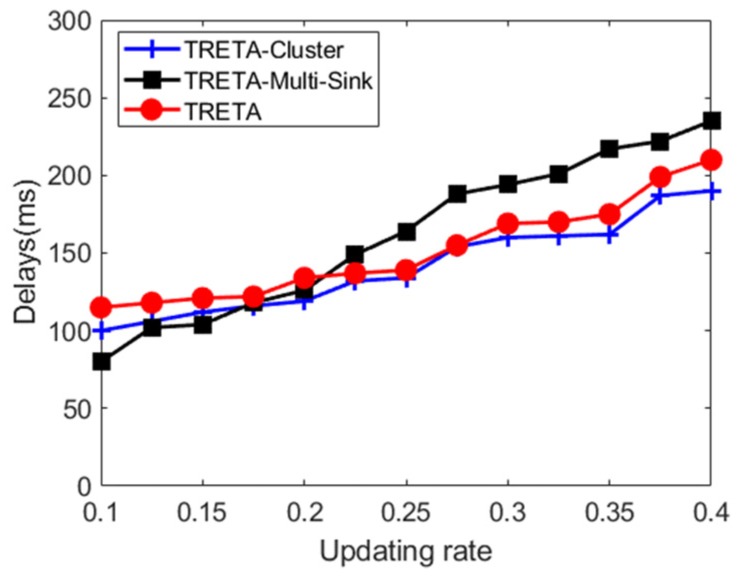
Delay in task assignment under different network update rates.

Figure 10 shows the delays of the three proposed strategies for different network update ratios. As the network update ratio increases, the delays in the three strategies increase. Under the current network simulation environment, analysis shows that the delays of TRETA-Cluster and TRETA-Multi-Sink are smaller than TRETA when the network update ratio is lower than 25%, which is due to the fact that TRETA-Cluster and TRETA-Multi-Sink have a small number of updates in the network, they are processed locally without affecting the reliability of the task target, and a complete network update is not required. Nevertheless, when the network update ratio exceeds 25%, the delay of TRETA-Multi-Sink increases rapidly and becomes higher than TRETA, which is caused by the operation of ensuring global view consistency at such a high update rate.

(E) Energy Resilience of the Transmission for Task Assignments

**Figure 11:**
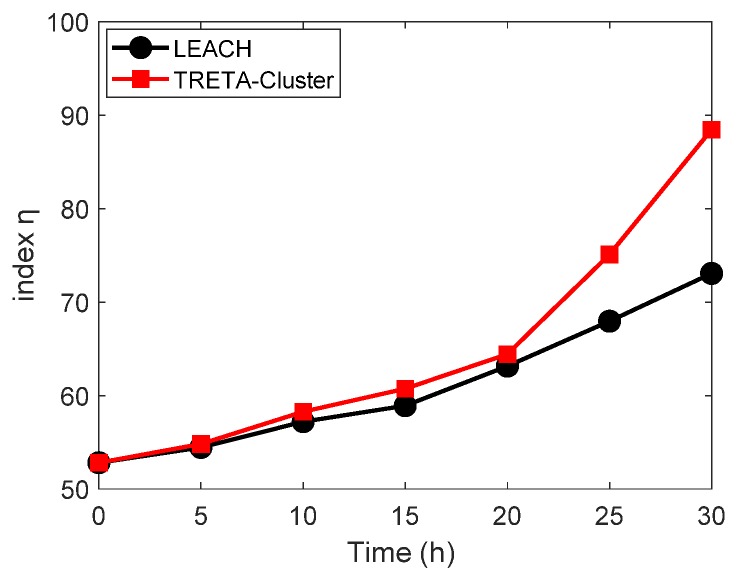
Energy resilience index of TRETA-Cluster and LEACH.

In order to evaluate the energy resilience of the transmission in WSNs, we quoted an index η in [34]. Since TRETA-Cluster is a distributed modification of TRETA for clustering topologies, we compared TRETA-Cluster and LEACH (Low Energy Adaptive Clustering Hierarchy) with index η. Simulation experiments were carried out under clustering topology. Let the number of initial active nodes be 500. For the same transmission task, we sample and count the number of packets received by sink nodes and the number of dead nodes at different time points. Then, we use Equation (22) to calculate the index η. Figure 11 shows the variation of the index η of the two algorithms over time. It can be seen from Figure 11 that the index η of both methods increases with time. Compared with LEACH, the growth rate of index η of TRETA-Cluster is more prominent, since TRETA-Cluster adds more redundancy packets than LEACH to meet the target reliability.

## 7. Concluding Remarks and Future Work

To solve the problems of poor scalability and reliability, significant communication overheads and delays in centralized task assignment for WSNs, we propose two distributed reliable and efficient transmission task allocations for WSNs, namely TRETA-Cluster and TRETA-Multi-Sink. The performance of these strategies is analyzed and evaluated, then compared with the centralized task assignment TRETA.

In TRETA-Cluster, the cluster head can perform local task assignment according to the stage target reliability constraint that reduces the communication cost and delay compared with TRETA. To summarize, the advantages include: (1) the cluster head can accurately select the number of nodes and the specific node ID in the cluster to complete the task, thereby saving energy; (2) as the topology changes occur in the cluster, the cluster head can select the replacement node according to the stage target reliability which has adaptive features. The advantages of TRETA -Multi-Sink are two-fold: (1) the global view is generated by merging the local views of multiple sinks that reduce the delay in obtaining the global view compared to the centralized acquisition method, and (2) multiple sinks respond to the local task assignment requirements in a globally optimized manner and do not need to communicate with remote nodes. It shows better scalability as well as overcoming the shortcomings of centralized task assignment in large-scale WSNs with significant communication overheads and delays.

From the analysis of both theoretical and simulation results, we show that the proposed distributed task allocation strategies are promising and superior to the centralized task allocation under the same network environment in terms of energy consumption, delay, and success rate.

From the observations of the limitations in distributed strategies when the network update ratio is higher than 25%, as TRETA-Multi-Sink no longer has the lead (which is caused by ensuring the consistency of the global view), we target the re-design and improvements of TRETA-Multi-Sink as a future direction to improve its efficiency. Another direction of investigation is to apply and adapt the proposed strategies in Cluster of Things (CoT) and Edge environments, where the communication conditions and environments highly yet dynamically vary all the time. Finally, this paper only researches the situation where the global view does not have concurrent updates. The efficiency of the distributed task allocation strategy when the network is concurrently updated will also be included as a future work direction.

## Figures and Tables

**Table 1:** Main simulation parameters.

Parameter	Value
Bandwidth	250 kbps
$e_{elec}$	50 nJ/b
$\varepsilon_{amp}$	$100\;\mathrm{pJ}/b/m^{2}$
Maximum transmission range of the node	50 m
The initial energy of the node	2 kJ
$e_{j}$	0.05 nJ/b

**Table 2:** Task parameters to be assigned.

Parameter	Value
Transmission direction	Upstream
Task interval	10 s
Task duration	[100 s, 300 s]
Target reliability	90%
Packet size	500 bytes
Number of packets per task	[100, 1000]
Packet loss rate	0–10%
Topology change rate	0–10%

## Nomenclature

**Symbol**	**Description**
$s_{i}$	*i*-th source node
$m_{i}$	The task to be assigned, which is the element in the set M(t)
$e_{ij}$	Energy consumed by the task $m_{i}$ on the node $N_{j}$
$e_{ij}^{comp}$	Calculated energy consumption of $N_{j}$
$e_{j}$	Average processing energy consumption of the nodes in the network
$t_{ij}^{comp}$	Calculation time of the task $m_{i}$
$e_{ij}^{comm}$	Communication energy consumption of the node $N_{j}$
$e_{ij}^{t}$	Energy consumption of transmitting data of length $l_{ij}$ bit at the distance d
$e_{elec}$	Energy consumption of operating the radio model for each bit
$\varepsilon_{amp}$	Coefficient of the transmit amplifier
$e_{ij}^{r}$	Energy consumption of receiving data of length $l_{ij}$ bit at the distance d
$P_{i}$	Task period of $m_{i}$
$S_{i}$	Start time of task $m_{i}$
$C_{i}$	Deadline for task $m_{i}$
$R_{WSN}$	Mission reliability of the network
$R_{s}$	Target reliability
$ClusterR_{s_{i,}sink}\left(t,i\right)$	*i*-th transmission Reliability of clustered WSNs from $s_{i}$ to the sink at time t
$R_{A}^{c}\left(t,i\right)$	Reliability of the *i*-th transmission performed by cluster *c*.
$R_{T}^{c}\left(t,i\right)$	Reliability of the head of cluster *c* which successfully sends the collected data of *i*-th transmission to sink at time *t*.
$MeshR_{s_{i},sink}\left(t,i\right)$	*i*-th transmission reliability in mesh WSNs from $s_{i}$ to the sink at time t
$R_{s_{i},sink}^{i}\left(t,i\right)$	Probability that there exists at least one path whose packet delivery rate is greater than the threshold of *i*-th transmission in the disjoint minimal path sets (from $s_{i}$ to sink)
$R_{path\left(s_{i},sink\right)}^{j}\left(t,i\right)$	*i*-th transmission reliability in mesh WSNs of *j*-th path from $s_{i}$ to the sink at time t
TG(V,E)	Physical topology of the network
RT(S, Ps)	Routing topology of the network
M(t)	Task set to be assigned at time t
$\varphi (m_{i})$	Mapped function of $m_{i}$
$Eng(\varphi (m_{i}))$	Energy consumption of the task $m_{i}$
$RT_{b}\left(S,\;Ps\right)$	Physical topology of the backbone network
$TG_{b}\left(S,Ps\right)$	Routing topology of the backbone network
$TG_{c}\left(S,Ps\right)$	The physical topology of the intra-cluster
$RT_{c}\left(S,Ps\right)$	Routing topology of the intra-cluster
$TG_{b^{\prime }}\left(V,E\right)$	Physical topology after backbone network update
$RT_{b^{\prime }}\left(S,Ps\right)$	Routing topology after backbone network update
$TG_{c^{\prime }}\left(V,E\right)$	Physical topology of the intra-cluster after update
$RT_{c^{\prime }}\left(S,Ps\right)$	Routing topology of the intra-cluster after update
$TG_{j}\left(V,E\right)$	Local physical topology of the *j*-th sink in a multi-sink topology
$RT_{j}\left(S,Ps\right)$	Local routing topology of the *j*-th sink in a multi-sink topology
$sink_{j}$	The closest sink to the task $m_{i}$
$sink_{jn}$	Sink adjacent to $sink_{j}$
$sink_{k}$	Sink involving topology changes
viewID	The version ID of the global view
$PathSet_{m_{i}}$	Path set of the source node to the sink in the coverage area of the task $m_{i}$
${PathSet_{m_{i}}}^{\prime }$	Path set after deleting the paths with link conflict in $PathSet_{m_{i}}$
$Paths_{i}$	Transmission path of each source node $s_{i}$ to sink in ${PathSet_{m_{i}}}^{\prime }$
$R_{s_{i}}$	Reliability of $Paths_{i}$
$E_{s_{i}}$	Energy consumption of $Paths_{i}$
$path_{R_{s}}$	Path to meet the target reliability $R_{s}$
$R_{CH}$	Target reliability of cluster head to sink after reliability allocation
$R_{CH^{\prime }}$	Updated $R_{CH}$
$R_{CM}$	Target reliability of cluster head after reliability allocation
$sink_{k}^{res}$	Sink respond to update request of $sink_{k}$
$sink_{k}^{res}.RT_{j}\left(S,Ps\right)$	Routing topology of the j-th sink in $sink_{k}^{res}$
$sink_{k}^{res}.TG_{j}\left(V,E\right)$	Physical topology of the j-th sink in $sink_{k}^{res}$
$sink_{k}.record$	Update operation record of $sink_{k}$
$sink_{k}^{res}.log$	Log of sink which respond to update
$S_{W},\;S_{R}$	Quorum set of writing and reading
$P_{overlap}$	Overlapping probability of $S_{W}\;\mathrm{and}\;S_{R}$
CM	Communication load
$Num_{cp}$	Number of packets in the allocation policy by multicast,
$Num_{gv}$	Communication load for transmitting one global view
WM, RW	Writing load, reading load
VUM	Write load of version and update operation of the global view when it is updated
$RT_{d\_c}$	Delay of task assignment for the clustered topology
$RT_{c}^{\prime }$	Delay of the sink allocates a task to the subgraph composed of the cluster heads
MC	Delay of the multicast performed by the sink
CHD	Delay in the allocation of the task by the selected cluster head
$RT_{d\_d}$	Delay of TRETA-Multi-Sink
$\bar{RT_{c}^{\prime \prime }}$	Average of the delays of the tasks assigned by multiple sinks locally
$C_{rt}$	Time to ensure the final consistency of the view
$W_{rt}$	Time of writing the global view whenever it is updated
$R_{rt}$	Time of reading the global view when the distributed task is assigned
η	Index for energy resilience of the transmission
$N_{p}$	Number of packet received by the sink after fixed time
S	Number of initial active nodes
D	Number of nodes death after a fixed time
